# A compendium of horizontal gene transfers in Metazoa

**DOI:** 10.1038/s41597-026-07354-5

**Published:** 2026-05-04

**Authors:** Johnathan A. Spaulding, Janna L. Fierst

**Affiliations:** Biomolecular Sciences Institute and Department of Biological Sciences, Florida International University, 11200 8th Street, 33199 Miami, FL USA

## Abstract

With more eukaryotic genomes available for study researchers have been able to identify a growing number of horizontal gene transfer (HGT) candidates. We compiled 9,495 protein coding genes that were identified as horizontally transferred to metazoan hosts in the published literature. This dataset contains gene transfers from bacteria, fungi, archaea and protists to metazoans. We assigned a confidence score to each gene based on the methods used in the scientific paper reporting HGT. All the coding sequences and protein sequences for the HGT genes are stored in a fig share repository. This dataset can be used to identify trends in genome and protein evolution and provide a foundation for creating a centralized HGT database for eukaryotes.

## Background

Horizontal or lateral gene transfer (HGT) is the movement of genetic material (DNA) from one organism to another, separate from parent to offspring^1^. This mode of genetic transfer was first discovered in bacteria and accounts for a significant amount of microbial genetic diversity^2^. Interestingly, the genes that are transferred to recipient bacteria often perform biological functions that give the recipient an evolutionary advantage^3^. For instance, bacteria that are susceptible to antibiotics can acquire antibiotic resistance genes via HGT from antibiotic resistant bacteria in the area^2^. This leads to an expansion in the ecological niche for the newly antibiotic-resistant bacteria, illustrating the importance of HGT.

It is important to note that there are other mechanisms of HGT. For instance, bacteria can perform HGT in three different ways. The first mechanism previously described is called conjugation in which a host bacteria forms a conjugation pilus with the recipient bacteria, which effectively creates a bridge that facilitates the host bacteria to transfers genes to the recipient bacteria^4^. The other mechanism is called transformation in which bacteria can take in DNA from the environment into the bacterial cell^5^. The third mechanism is called transduction in which a bacterial phage contains DNA from another bacteria and injects this DNA into the recipient bacteria^6^. While the bacterial mechanisms of how HGT occurs is known, the mechanisms by which HGT occurs in eukaryotes is poorly understood^7^. However, we do have examples of HGT occurring in eukaryotes. For example, organisms that have endosymbionts have been found to have genes that originated from the endosymbiont, which is known as endosymbiotic gene transfer (EGT)^8,9^. This mode of gene transfer is well known and important in eukaryotes since multiple genes from the mitochondria and chloroplast have been transferred to the host cell nucleus, hence impacting eukaryotic evolution^10–12^. Furthermore viruses have been shown to transfer genes to other organisms as well as transposons^13^.

HGT is an established phenomenon in both prokaryotes and eukaryotes, yet its prevalence differs markedly among eukaryotic lineages. Whereas plants, fungi, and protists have been shown to acquire foreign genes at comparatively higher rates, HGT events in metazoans remain infrequent and are considered exceptional rather than commonplace^14–16^. The scarcity of verified HGTs in metazoans could be due to technological or biological reasons. Technologically, there could be sequencing bias, in which organisms that are typically sequenced aren’t the organisms receiving HGTs^17^. Biologically, Metazoan organisms may be less prone to acquire HGTs due to the physical separation of germ-line cells from somatic cells. Somatic cells may shield the germline from foreign DNA, decreasing the chance of foreign DNA being transmitted to the progeny^18^. Thus, HGT occurring in metazoans will be less likely while HGT occurring unicellular eukaryotes, plants and fungi that lack this barrier will have a higher frequency^19^.

While metazoans may not engage in HGT as frequently as prokaryotes, what they do have in common is that transferred genes can provide adaptive functions potentially aiding the recipient. For example, there have been multiple cases of plant parasitic nematodes gaining foreign genes from bacteria and fungi that facilitate parasitism^20^. The plant parasitic nematode *Bursaphelenchus xylophilus* has gained multiple genes from fungi that have aided it in parasitism such as plant cell wall degrading enzymes^21^. To identify more cases of eukaryotic HGT and study its biological significance, more eukaryotes from neglected groups need to have their genomes and transcriptomes sequenced and analyzed^17,22^.

Advances in machine learning and statistical methods could form the foundation for rigorous bioinformatic identification of HGTs, but methods development has been slowed by a lack of validated HGTs^23^. In the absence of a comprehensive empirical dataset researchers developing HGT identification methods have used simulated HGTs. This frequently involves creating synthetic chimeric genomes by inserting known foreign DNA sequences into the recipient genome at random, with the goal of testing a novel method to identify HGT in genomes^23,24^. For example, Jaron *et al*. simulated HGT by randomly selecting and replacing DNA sequences across 10 genomes, then applied their new method, SigHunt to identify the inserted sequences^24^. Simulations may approximate very recent HGTs but over time these sequences will be subject to mutation and selection, changing the foreign origin ‘signal’ HGT identification methods rely on.

Fortunately, biology is experiencing an unprecedented increase in the amount of data being generated^25,26^. The cost of sequencing genomes has decreased for the last two decades^27^. This, in turn, has enabled more laboratories to generate rich genomic datasets^28^. For prokaryotic organisms, curated databases such as HGTree2.0 are available that catalogue horizontally transferred gene sequences alongside relevant biological information^29^. In contrast, no equivalent curated database exists for eukaryotic HGT. Despite the growing body of literature reporting HGT events across phylogenetically diverse eukaryotic lineages, a comprehensive resource consolidating these findings from both past and current publications remains absent. A major bottleneck in studying HGTs stems from the fact that this information, although growing, remains confined to individual publications.

In this study, we created a dataset that contains 9,495 HGT candidates identified in published articles between 2000 and 2025. This dataset contains HGTs from bacteria, fungi, archaea, and protists to metazoan recipients. We also provide both tested and untested predictor variables for the transferred genes which may be used to statistically differentiate HGTs from native sequences. Our tested predictor is Guanine-Cytosine (GC) content, while our untested predictors are amino acid length and coding sequence length^30^. This dataset will allow researchers to comprehensively analyze sequence data, discover emergent patterns of HGTs and provide a foundation for creating a centralized HGT database for metazoans.

## Methods

### Workflow for metazoan HGT dataset (mHGT) paper search

To create the mHGT dataset, we identified articles using a Web scraper program called ScrapPaper that extracts information from PubMed search results^31^.The program extracted the titles of all research papers in the published literature containing the keywords “ Lateral Gene Transfer”, “Horizontal Gene Transfer”, and “metazoan”(https://pubmed.ncbi.nlm.nih.gov/?term = Lateral + Gene + Transfer%2C + Horizontal + Gene + Transfer%2Cmetazoan&filter = years.2000-2025) from 2000 to 2025. The output of this program displayed a dataset containing titles of research papers identifying prokaryote/fungi/archaea to metazoan transfers, prokaryote to prokaryote transfers, and viral to metazoan transfers. Only the research papers pertaining to bacteria-, fungi-, archaea-, and protist-to-metazoan transfers were used to create the mHGT dataset. Thus, out of the 151 HGT papers that were identified using ScrapPaper only 36 papers were included in the mHGT dataset^20,32–66^.

**Fig. 1:**
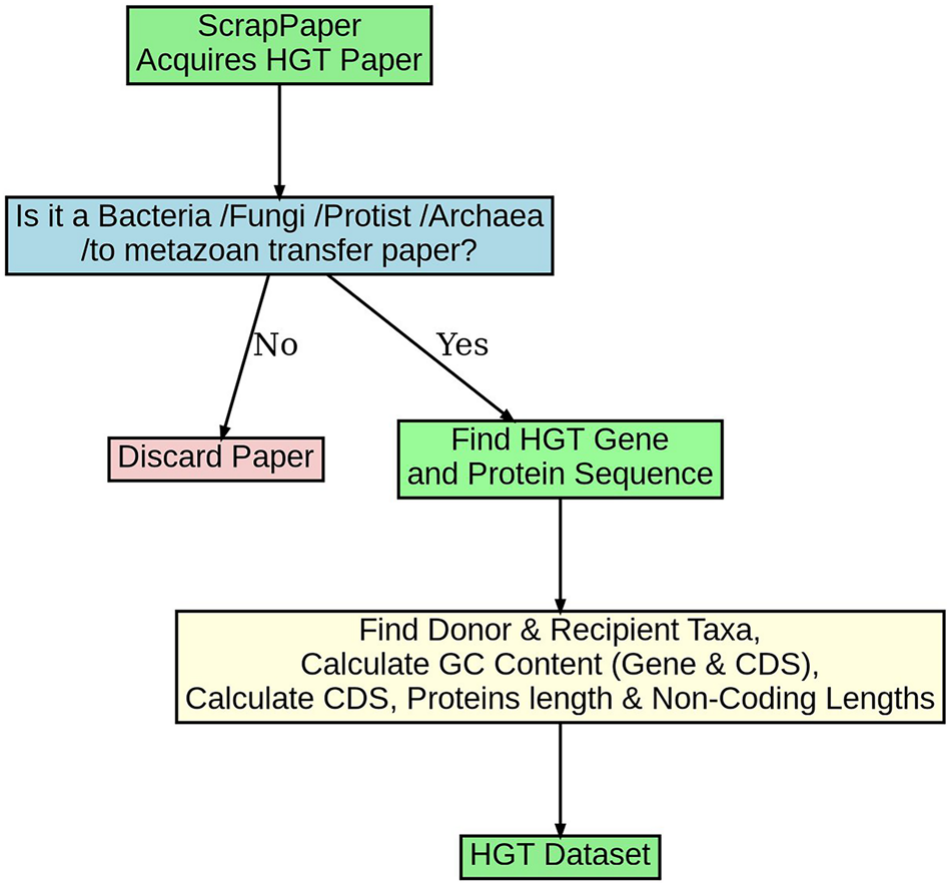
Data collection workflow for the mHGT dataset. Each paper was selected based on the type of HGT. The mHGT dataset focuses on bacteria-, fungi-, archaea-, and protist-to-metazoan transfers. Once the paper was selected the next step was to locate the gene and protein sequences.

For each article the transferred nucleotide gene sequence and corresponding amino acid sequence were obtained from the National Center for Biotechnology Information (NCBI) or the data deposition website or database reported by the authors. The transferred gene accession number, genome assembly ID, protein accession number, gene name/function, phylogenetic support, confidence score, inferred donor taxon (Bacteria, Fungi, Protist, or Archaea), host taxon, and paper reporting the HGT(s) were also recorded when possible. Custom Python scripts, available at https://github.com/Genome-explorer/A_compendium_of_the_metazoan_horizontal_gene_transfer_dataset were used to calculate the Guanine-Cytosine percentage, gene length, protein length, and coding sequence length for each of the HGTs. An outline of our workflow is displayed in Fig. 1.

### Confidence score metric

We created a confidence score metric to assess the likelihood of the reported gene transfer being a true HGT. Both errors in wet lab procedures and sequencing can lead to false positive HGT identification. Experimentally, multiple steps in the wet lab including DNA extraction, library construction, amplification of DNA and sequencing can introduce errors^67^. Downstream effects of these processes can lead to gene prediction errors including false sequences that are not present in the organism^68^. This may reflect either genomic contamination by foreign DNA during the wet lab procedures, or homotype false duplications arising from accumulated sequencing errors that produce under-collapsed sequences in the genome assembly^69–72^.

To increase confidence that a gene is a true HGT, multiple methods should be used to assess both presence in the genome and evolutionary origin^72^. Thus, our confidence score ranges from 1 to 5, with higher scores indicating greater certainty in the gene transfer event (Table 1). Each gene is assigned a confidence score based on multiple lines of evidence provided in the original paper reporting the transfer event. Both empirical laboratory evidence and computational analyses were weighted depending on reliability of the techniques and potential biological information. For example, papers that included the use of both *in situ* hybridization (or RNAi or Crispr) and phylogenetic analysis have a confidence score of five. *In situ* hybridization is a molecular technique that shows the location of a specific sequence of DNA in the biological sample, which excludes the chance that the gene is a result of contamination^73^. Phylogenetic analysis is a reliable statistical method for identifying evolutionary gene origin and confirming sequences have been horizontally transferred^74^.

**Table 1:** Confidence scores and the required conditions for each score.

Confidence Score	Criteria	Evidence Description
5	*In situ* hybridization + Phylogenetic analysis	Confirms gene location, excludes contamination; phylogeny supports HGT origin
4	RNA validation + Phylogenetic analysis	Gene is expressed; phylogenetic analysis supports evolutionary history
3	PCR or Southern blot + Phylogenetic or sequence-based analysis	Confirms presence (with contamination risk); phylogeny or sequence analysis adds reliability
2	Sequence-based method + Phylogenetic analysis	Sequence similarity + phylogeny
1	Only one method (e.g., sequence or phylogeny)	Only one line of evidence is used

Higher confidence scores indicate greater certainty that the gene represents a true horizontal transfer event.

A confidence score of four was assigned to studies in which empirical RNA-based evidence — derived from approaches such as quantitative PCR, expressed sequence tags (ESTs), or the RACE method — and was corroborated by phylogenetic or sequence-based analysis^75–77^. RNA evidence demonstrates that the gene is actively expressed in the organism^78^. Research papers using both empirical DNA-based methods — including Polymerase Chain Reaction (PCR), Southern blotting or DNA probes^79^,— and phylogenetic or sequence-based analysis^80^ have a confidence score of three. While DNA-based methods such as PCR can confirm gene presence in biological samples, contamination poses a significant risk of false positives, as amplified genes may originate from contaminant organisms rather than the target species^81^. Papers that used both a sequence-based method^80^ such as BLAST (Basic Local Alignment Search Tool) and phylogenetic analysis were assigned a confidence score of two. Lastly, papers that only use one method of detection such as sequence-based methods or phylogenetic analysis or empirical DNA-based methods or RNA-based methods were assigned a confidence score of one.

## Data Overview

### Recurrent top 7 phyla that experienced HGT

**Fig. 2:**
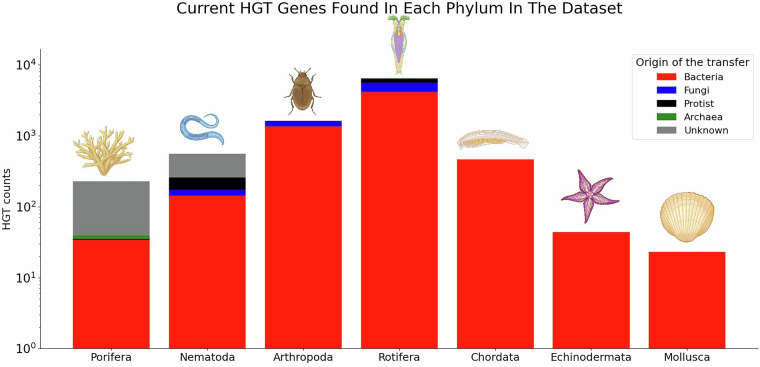
The number of HGTs found in each of the top 7 phyla in the mHGT dataset. ‘Unknown’ are genes with origin not explicitly stated in the article^85^.

**Fig. 3:**
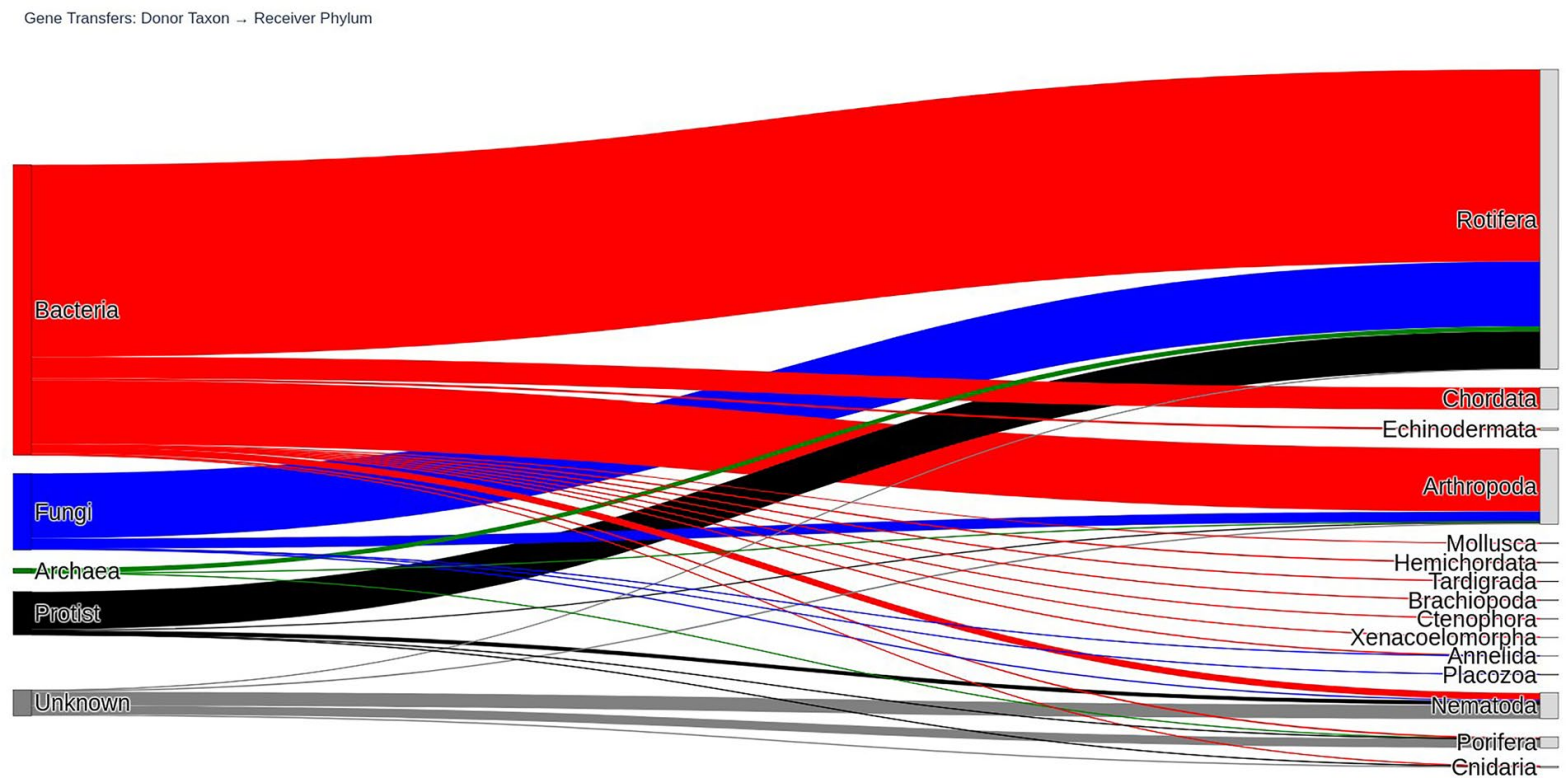
Sankey diagram showing the flow of gene transfers from donor organism to the 13 phyla present in the dataset.

**Fig. 4:**
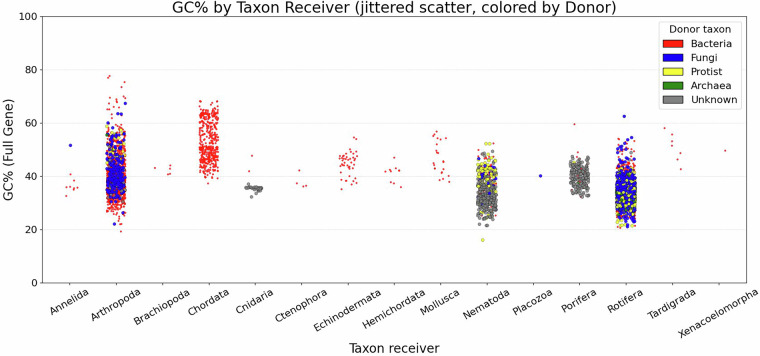
The GC (as % of the gene sequence) for all transferred genes in each phyla. The data are plotted with a slight displacement (jitter) to enhance visualization.

After identifying horizontally transferred genes present in different organisms, we analyzed the distribution patterns within each recipient phyla. The top seven phyla that experienced the most gene transfers were: Rotifera, Arthopoda, Nematoda, Chordata, Porifera, Echinodermata, and Mollusca. This was calculated by the number of transferred genes present in each species that belong to their respected phyla. Phylum Rotifera had the highest number of transferred genes out of all the phyla present in the dataset (Fig. 2). The phyla with highest number of transferred genes with unknown origin were Nematoda (n = 298) and Porifera (n = 190; note-, the log scale makes the unknown bar in Porifera appear larger than the Nematoda unknown bar). These transferred genes had a non-metazoan origin reported in the paper, but the authors also reported uncertainty in assigning the donor organism. Phylum Mollusca received the fewest HGTs of these 7 phyla (Fig. 2). Overall, each of the top 7 phyla had the most foreign genes originating from bacteria. In total 13 phyla were represented in the mHGT dataset. The phyla containing the most bacterial gene transfers were Rotifera, Arthopoda, Chordata, and Nematoda respectively (Fig. 3). Genes with fungal origins were abundant in Rotifera and Arthopoda. Moreover, Rotifera and Nematoda contained the most genes with protist origins. Overall, GC content across HGT in the dataset typically ranges between 17% and 78% (Fig. 4). This is in contrast to metazoan genomes for which GC content frequently falls between 35% and 55%^82,83^.

## Data Records

The mHGT dataset is available at: Metazoan horizontal gene transfer dataset a compendium^84^. The Figshare repository contains three main files: Global_x36_rotifer_nematode.csv, online_repository.tar.gz, and Readme_please_read.txt. The Global_x36_rotifer_nematode.csv file contains the complete mHGT dataset described in this paper. The online_repository.tar.gz is a compressed archive containing 36 numbered folders, each corresponding to a single source publication used in the dataset. Each folder comprises: a fastaFiles sub folder containing full gene, coding sequence (CDS), and protein sequences in FASTA format; a Final_horizontal_gene_transfer_dataset.csv file with all HGT genes identified from that specific paper; a cumulative Global CSV file combining data from all previous papers, and where applicable, a Readme.txt file with paper-specific details and notes. The folders all_CDS, all_full_gene, and all_protein contain all FASTA files in numerical order, along with combined FASTA files (all_CDS.fasta, all_full_gene.fasta, and all_protein.fasta) generated from those sequences. The archive also includes two additional CSV files: original151_articles_16Pages_5-21-2025.csv, which contains all horizontal gene transfer papers identified using the ScrapPaper program, and allSelectedPapersForDataset_5-21-2025.csv, which contains the subset of papers selected for inclusion in the mHGT dataset. In the dataset each row corresponds to a single gene that was transferred from a donor organism to recipient organism. The columns in the dataset are:

1. Gene_Identification_number(Genome_Exclusive): records identifiers for sequences that exist only within the context of a specific genome assembly and have no independent NCBI record. The identifiers are labeled how they are reference in the paper or genome assembly. Fields that do not apply to a given entry are denoted N/A.
2. Gene_Identification_number(Paper_Exclusive): records identifiers for three categories of sequence: those reported exclusively within the source publication without deposition in a public database; those deposited in non-NCBI repositories; and those for which an NCBI record exists but encompasses a multi-gene genomic fragment, requiring positional coordinates to unambiguously identify the gene of interest. Fields that do not apply to a given entry are denoted N/A.
3. Gene_accession_number(From_NCBI): records standard NCBI nucleotide accessions for sequences retrievable by direct database lookup.
4. Genome assembly ID: Represents the genome version used in the paper.
5. Protein_Identification_Number(Paper_exclusive): records protein identifiers from non-NCBI sources.
6. Protein_accession_number(From_NCBI): The NCBI accession number of the protein or accession number of the protein in a genome assembly.
7. Gene_name/Function: The name of the gene or its function, when available.
8. Full_Gene_Length: The full length of the gene sequence.
9. Coding_Sequence_length (CDS): represents the combined theoretical maximum across all exon sequences, and that the protein length reflects the largest theoretical protein product.
10. Non_coding_Sequence_length: The number of nucleotides that are not coding for the protein sequence such as 5′UTR, 3′UTR, and intron. This value was calculated by subtracting the CDS length from the full gene length.
11. Amino_Acid_sequence_length (aa): Represents the largest theoretical protein product from the amino acid sequence length.
12. G + C_content_full_gene: The decimal fraction of the amount of Guanine-Cytosine in the full gene sequence. The formula: GC Content % = Number of G’s and C’s divided by the total number of bases in the sequence multiplied by 100. If any of the genetic sequences have an “N” in the sequence, the “N” is excluded from the calculation.
13. G + C_content_CDS: The decimal fraction of the amount of Guanine-Cytosine in the CDS gene sequence.
14. Phylogenetic support: Identity’s whether this gene was shown to be transferred using a phylogenetic tree from the paper that identified the gene.
15. Confidence Score (1–5): A certainty metric used to assess the likelihood of gene transfer.
16. Taxon_Donor (Bacteria, protist, fungi): The taxon of the Donor organism of the gene that was transferred.
17. Taxon_receiver (By_Phylum): The taxon of the recipient organism that received the gene.
18. Receiver_Genus_species: The organism that received the transferred gene.
19. PMID: The unique paper identifier that is associated with the paper that presented the transferred gene. The PMID identifier will be used for each paper.
20. Year: The year the paper was published.
21. Paper: The title of the paper that presented the transferred gene.

Lastly, the codes used in this study are available https://github.com/Genome-explorer/A_compendium_of_the_metazoan_horizontal_gene_transfer_dataset.

## Technical Validation

After the Web scraper program called ScrapPaper extracts information from the PubMed search results, it outputs a dataset containing HGT papers containing the keywords “Lateral Gene Transfer”, “Horizontal Gene Transfer”, and “metazoan” from 2000 to 2025^31^. The total amount of HGT papers in the dataset was 151, but it was filtered down to 36. During the filtering process only HGT papers about bacteria-, fungi-, archaea-, and protist-to-metazoan transfers were kept in the final dataset. In addition, the selection of transferred genes was performed by reading each of the source publications, and including genes explicitly identified as horizontally transferred by the original authors. For the publications that reported multiple HGT candidates all the sequences were identified and recorded. All pseudogenes identified as HGT candidates in the source publication were excluded from the dataset, since these non-functional genes will not provide a benefit to the host species. For each included sequence we verified that the reported identifier referred to the correct organism that was described in the source publication, with increased emphasis on the correct genome assembly version where applicable. For the sequences deposited in NCBI, the accession records were checked for suppression status, and any suppressed accessions were excluded from the dataset. Where ambiguity remained regarding sequence identity or taxonomic assignment for the recipient species, the entry was excluded to maintain dataset integrity. All papers used to create this dataset have been peer reviewed and published in their respective journals.

## Data Availability

The proposed dataset is available at our Figshare repository: 10.6084/m9.figshare.30408499^84^. The three main files in the repository are Global_x36_rotifer_nematode.csv, online_repository.tar.gz, and Readme_please_read.txt. The complete mHGT dataset is present in the Global_x36_rotifer_nematode.csv file. An archive file called the online_repository.tar.gz contains 36 numbered folders that each contain a dataset that corresponds to the source paper that provided the data.

## Code availability

Code for the Python scripts that were used to calculate the Guanine-Cytosine percentage, protein length, and coding sequence length for each of the transferred genes is available at https://github.com/Genome-explorer/A_compendium_of_the_metazoan_horizontal_gene_transfer_dataset. The code for the web scraper is available at https://github.com/rafsanlab/ScrapPaper.
